# Emerging Therapies for the Treatment of Atherosclerotic Cardiovascular Disease: From Bench to Bedside

**DOI:** 10.3390/ijms24098062

**Published:** 2023-04-29

**Authors:** Marko Kumric, Hrvoje Urlic, Josko Bozic, Marino Vilovic, Tina Ticinovic Kurir, Duska Glavas, Dino Miric, Jaksa Zanchi, Anteo Bradaric-Slujo, Mislav Lozo, Josip A. Borovac

**Affiliations:** 1 Department of Pathophysiology, University of Split School of Medicine, 21000 Split, Croatia; marko.kumric@mefst.hr (M.K.);; 2 Department of Endocrinology, Diabetes and Metabolic Diseases, University Hospital of Split, 21000 Split, Croatia; 3 Cardiovascular Diseases Department, University Hospital of Split, 21000 Split, Croatia

**Keywords:** atherosclerosis, cholesterol, inflammation, therapy, statins, bempedoic acid, PCSK9 inhibitor, omega-3

## Abstract

Primarily a consequence of sedentary lifestyle, atherosclerosis has already reached pandemic proportions, and with every year the burden of it is only increasing. As low-density lipoprotein cholesterol (LDL-C) represents a crucial factor in atherosclerosis formation and progression, stringent lipid-lowering therapy could conceivably be the key to preventing the unfavorable outcomes that arise as a consequence of atherosclerosis. The use of statins in lipid-lowering is often burdened by adverse events or is insufficient to prevent cardiovascular events as a monotherapy. Therefore, in the present review, the authors aimed to discuss the underlying mechanisms of dyslipidemia and associated atherosclerotic cardiovascular disease (ASCVD) and preclinical and clinical trials of novel therapeutic approaches to its treatment, some of which are still in the early stages of development. Apart from novel therapies, a novel change in perspective is needed. Specifically, the critical objective in the future management of ASCVD is to embrace emerging evidence in the field of atherosclerosis, because clinicians are often burden by common practice and personal experience, both of which have so far been shown to be futile in the setting of atherosclerosis.

## 1. Introduction

Atherosclerosis is a chronic inflammatory disease of the large and medium-sized arteries’ subintimal layer, and it results in the formation of fibrofatty lesions in a process mediated by the interaction of various well-established risk factors, such as hypercholesterolemia, hypertension, diabetes mellitus, smoking, and many others [1,2,3]. As a result of sedentary lifestyle, atherosclerosis has reached pandemic proportions, and with every year the burden of it is only increasing. The clinical manifestations of atherosclerosis: coronary artery disease (CAD), cerebrovascular disease, and periphery artery disease (PAD) actually represent the main cause of death and morbidity in the world [4,5]. Although its high prevalence and significant healthcare burden have pushed us toward exploration of this disorder, its pathophysiology has still not been completely elucidated. The largest gap in knowledge is in the treatment aspect, as we still cannot treat atherosclerosis with sufficient success, and this commonly limits us to the treatment of its consequences. According to leading experts in the field, we do possess the tools to mitigate atherosclerosis (through the targeting of very low lipid levels sufficiently and early), but we simply do not utilize them [6]. Apart from lifestyle interventions (such as smoking cessation, physical activity, and diet), the current cornerstone in atherosclerosis prevention is treating the dyslipidemia with statins and, to a lesser extent, with ezetimibe and proprotein convertase subtilisin/kexin type 9 (PCSK9) inhibitors [7]. Considering the suboptimal results of treatment and the side effects of statins underlying their intolerance, novel non-statin therapies have emerged in recent years. For instance, various recent approaches to immunotherapy and vaccination have shown promising results in restraining atherosclerosis in animal models. Accordingly, emerging lipid management studies extended beyond LDL-C reduction, putting alternative pathways into focus and thus bringing novel insights into atherosclerosis pathophysiology.

In the present review, the authors aimed to discuss the underlying mechanisms of dyslipidemia and associated atherosclerotic cardiovascular disease (ASCVD) and preclinical and clinical trials of novel therapeutic approaches its treatment some of which are still in the early stages of development.

## 2. Contemporary View on the Pathophysiology of Atherosclerosis

Although the detrimental cascade underlying the development of atherosclerosis in arteries is perhaps one of the most studied processes in humans, the reasons for the variability in terms of plaque progression and consequent outcomes still remain elusive [8]. The initial step in this process is endothelial cell (EC) activation, a process during which ECs switch from an anti-thrombotic and anti-inflammatory phenotype in which the endothelial monolayer is not susceptible to the binding of plasma leukocytes, to a phenotype identified by the increased expression of cell-adhesion molecules and marked synthesis of proinflammatory cytokines and chemokines, thus paving the way for the development of atherosclerotic plaques [9]. Although increased endothelial shear stress and proinflammatory cytokines usually trigger such activation, the fact that low-density lipoprotein cholesterol (LDL-C) plays a pivotal role in the early development of atherosclerosis needs to be addressed. A body of data supports that notion. 

To start with, Goldstein and Brown discussed that atherosclerosis would probably not occur in the first place if LDL-C levels were substantially low (10–20 mg/dL), and if we kept our LDL-C levels to those of neonates or of other animals including primates (<50 mg/dL), then atherosclerosis would probably be a marginal disease [10]. Evidence in patients with hypercholesterolemia who develop significant plaques at a very early age and the fact that experimental models of atherosclerosis are easily achievable using high-fat diet further support this hypothesis. Conversely, patients with a loss-of-function mutation in PCSK9, an enzyme that steers LDL-C receptors to lysosomes and thus reduces their cell surface expression, have very low incidence of major CV events, even when compared to patients treated with statins [11]. It is presumed that this difference is based on the duration of the LDL-C lowering, as patients with PCSK9 mutations have low LDL-C from a young age, whereas patients in which we prescribe statins have usually developed atherosclerotic processes, so more rigorous LDL-C lowering is needed in order to prevent a CV event [12]. In addition, after decades of exposure to borderline/slightly elevated LDL-C levels, the risk of atherosclerotic disease in the general population is as much as 3 to 4 times higher in contrast to the population with persistently low LDL-C levels [13]. Nevertheless, this should not discourage the use of statins in older age, as it has been shown that plaques can reduce in size following periods of lipid-lowering therapy [14]. Moreover, a recent SCOPE-75 trial showed that statins reduce all-cause mortality even if introduced in patients older than 75 years of age [15]. Another perspective to this issue is the fact that except for when LDL-C concentrations are extremely high or low, it is tremendously challenging to predict whether an individual will develop myocardial infarction or stroke [16]. According to a prevailing theory, LDL-C initiates plaque formation by penetrating through dysfunctional endothelium, where it is retained as a result of its propensity to bind to glycosaminoglycans [17]. Yet, although for many years we attributed this progression to the oxidized form of LDL-C based on animal models, limited evidence supports the causal role of oxidized LDL-C in humans, especially when one considers the therapeutic implications (intervention that targeted it all failed) [18]. Thus, although LDL-C is a major player in atherosclerotic plaque pathobiology, we should seek explanations beyond the existing oxidation hypothesis [19,20]. In simple terms, if we were to match all of the other risk factors, we would find that the higher the LDL-C values, the faster the plaques would the evolve. However, these other factors are never equal, and for a given level of LDL-C, plaque formation is also accelerated by other risk factors including hypertension, smoking, and diabetes mellitus. Aside from LDL-C, recent data imply that triglyceride-rich lipoproteins (TGRL) also participate causally in atherogenesis, and such findings are being therapeutically challenged [21].

Emerging evidence suggests that inflammation may represent a mechanistic link between dyslipidemia and traditional risk factors in the development of atherosclerosis. Such an association is supported by both epidemiological and experimental data. From an epidemiological perspective, high sensitivity C-reactive protein (hsCRP), a biomarker of inflammation, has been shown to predict CV outcomes independently of traditional risk factors in a diverse population regardless of the presence of manifest CV disease [22,23]. In fact, the risk related to a 1 SD increase in hsCRP is at least as great as that associated with a comparable increase in blood pressure or cholesterol, even when adjusted for multiple risk factors [22,23]. Despite the abundance of data, clinicians are commonly reluctant to consider hsCRP in this regard, stating that hsCRP is too variable, even though evidence implies that the tracking coefficients of hsCRP over time correspond to those of BP and cholesterol [22]. Both innate and adaptive immunity seem to play a role in the progression of atherosclerosis [24]. As we previously noted, the inflammatory process inside the intima starts as a result of endothelial activation that leads to the increased synthesis of a large array of cytokines and chemokines that serve as alarm signals for the recruitment of immune cells [25]. Recruited monocytes subsequently convert to macrophages and accumulate lipids, thus forming foam cells. Apart from innate immunity, adaptive immunity is also included in the pathophysiology of atherosclerosis. Specifically, T_H_1 lymphocytes were found to secrete pro-inflammatory cytokines and, in fact, to aggravate atherosclerosis, whereas T_H_2 and T_reg_ lymphocytes were found to produce anti-inflammatory cytokines and counteract the effects of T_H_1 [26]. Furthermore, novel evidence implies that hematopoiesis may promote atherosclerosis. Specifically, the presence of clonal hematopoiesis of indeterminate potential (CHIP), i.e., clones of leukocytes carrying driver genes for leukemia, has been independently associated with increased incidence of CV events [27]. This idea is supported by experimental evidence in rodent models in which mutations in genes such as *Dnmt3a, Tet2*, or *Jak2^V617F^* (all being mutations associated with CHIP) have been associated with accelerated atherosclerosis, the upregulation of pro-inflammatory pathways, and increased activity of the AIP2 and NLRP inflammasomes [28,29,30]. As sleep disturbance, mental stressors, and various infections are all able to stimulate hematopoiesis, it is viable that CHIP might be one of the missing links associating environmental factors, inflammation, and atherosclerosis development [31,32]. Nevertheless, it is worth noting that we are still unable to successfully predict which individuals with CHIP will develop ASCVD and/or leukemia [33].

## 3. Emerging Therapies in Atherosclerosis

**Figure 1:**
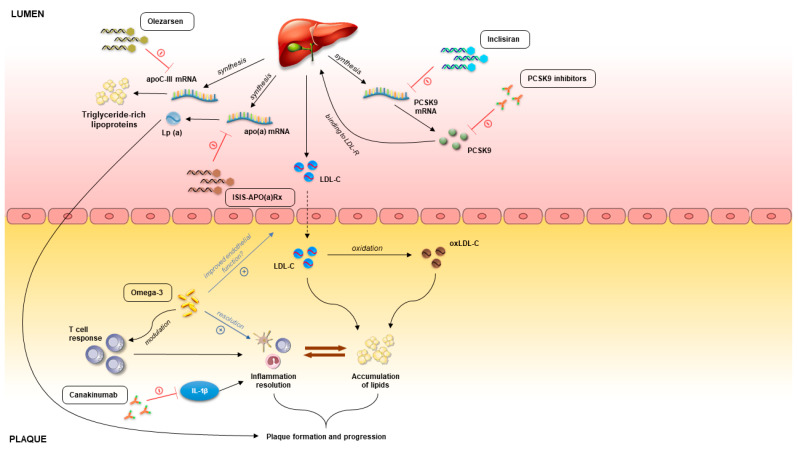
Pathophysiology of atherosclerotic plaque and molecular targets on novel atherosclerotic therapies. Abbreviations: PCSK9, proprotein convertase subtilisin/kexin type 9; oxLDL, oxidized LDL; LDL-R, LDL-receptor; LDL-C, LDL-cholesterol; LDL, low-density lipoprotein; Lp(a), lipoprotein(a).

**Figure 2:**
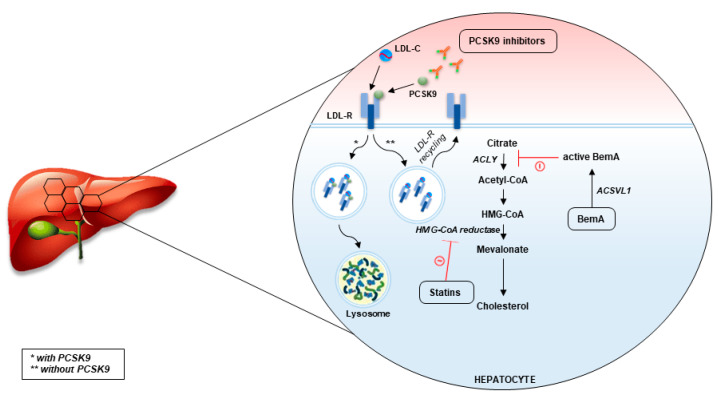
Mechanisms by which atherosclerotic drugs mitigate hyperlipidemia.

Statins are viewed as fundamentals of dyslipidemia management. However, their use is burdened by adverse events (and more commonly the fear of adverse events), most commonly statin-associated myopathy [34,35]. In addition, in a number of cases, statins do not reduce cholesterol sufficiently, despite treatment with maximally tolerated statin doses [36]. According to the contemporary guidelines issued by different professional societies worldwide, in these cases, it is advised to use ezetimibe and PCSK9 inhibitors [7,37]. As was previously mentioned, ezetimibe is already widespread in clinical use. It is an inhibitor of the Niemann–Pick C1-Like 1 (NPC1L1) transporter which is in control of cholesterol absorption in the small intestine [38]. Moreover, these channels have also been found in high concentrations in hepatocytes. Inhibition of NPC1L1 transporter impedes the reabsorption of cholesterol from the small intestine which leads to overall increased excretion of cholesterol from the body [39]. Ezetimibe monotherapy has shown promising results in the management of dyslipidemia [39]. Moreover, ezetimibe as an add-on therapy to statin, after three months of treatment, has displayed significant additional LDL-C lowering of 13–20% [40,41]. The additive effect of ezetimibe to patients receiving statins reflects the fact that NPC1L1 expression is increased as a form of compensatory mechanism in patients receiving statins [38]. Furthermore, the IMPROVE-IT trial, which studied 18,144 patients over a median period of 6 years, indicated an absolute risk reduction of 2% in the statin–ezetimibe group when compared to statin monotherapy in which the composite outcome consisted of cardiovascular death, non-fatal MI, non-fatal stroke, unstable angina requiring rehospitalization, as well as percutaneous coronary intervention ≥30 days after randomization in the trial [19]. The mechanisms of novel drugs targeting plaque development and progression are summarized in Figure 1 and Figure 2.

### 3.1. PCSK9 as Therapeutic Target

Unlike ezetimibe, PCSK9 inhibitors are emerging “players” in the field of atherosclerosis, and real-world data on their safety and effectiveness are still being accumulated. These drugs are designed as monoclonal antibodies that support the recycling of LDL-C receptors. Namely, by binding to LDL-C receptors, PCSK9 leads to the internalization and breakdown of LDL-C receptors in the lysosomes [42]. Thus, inhibition of PCSK9 leads to increased LDL-C receptor expression and, hence, larger uptake of LDL-C [43]. In addition, unlike statins, PCSK9 inhibitors seem to reduce lipoprotein(a)¸ an independent and a causal risk factor for ASCVD [44]. However, a recent meta-analysis argued that even though alirocumab diminished lipoprotein(a), this reduction did not independently correlate with the reduction in major adverse cardiovascular events (MACEs) more than what was already granted by the reduction in LDL-C cholesterol [45]. Finally, recent data indicate a role of PCSK9 in the metabolism of triglyceride-rich lipoproteins, a fact that might be relevant for patients with hyperlipidemia in the setting of diabetes mellitus [46,47]. The effectiveness of PCSK9 inhibitors in terms of lipid lowering and the improvement of ASCVD outcomes is supported by an abundance of data. PCSK9 inhibitors such as evolocumab and alirocumab have been shown to reduce LDL-C by 43–64% as add-on statin therapy [48]. Furthermore, in a Phase 2b, randomized controlled trial (RCT), MK-0616, an oral PCSK9 macrocyclic peptide inhibitor, was shown to reduce LDL-C by up to 60.9% upon 8-week treatment in a population with diverse ASCVD risk [49]. Moreover, PCSK9 inhibitors were shown to improve CV outcomes in multiple RCTs. Pivotal evidence of CV benefit emerged from the FOURIER (evolocumab) and ODYSSEY OUTCOMES (alirocumab) trials, both of which successfully reached the primary endpoint [50,51]. Moreover, a recent PACMAN-AMI trial showed that the addition of alirocumab biweekly to high-intensity statin therapy following myocardial infarction resulted in significantly greater coronary plaque regression (non-infarct-related arteries) after 52 weeks in comparison to placebo [52]. However, a recent meta-analysis concluded that alirocumab reduces and evolucumab increases all-cause mortality (RR 0.83 and 1.26, respectively) and thus, that alirocumab might provide the best protection with respect to all-cause mortality and reduced risk of serious adverse events, whereas evolocumab might prove optimal in the setting of myocardial infarction for secondary prevention in high-risk patients [53]. Although a concern was raised that PCSK9 inhibitors might affect neurocognition and the hypovitaminosis of fat-soluble vitamins as a result of unprecedentedly low LDL-C levels, accumulated safety data provided encouraging results [54,55].

Monoclonal antibodies are not the only therapeutic modalities by which PCSK9 has been targeted. Inclisiran, a first-in-class small interfering RNA (siRNA) molecule that inhibits the hepatic synthesis of PCSK9 by RNA interference, first drew attention during the ORION-1 trial [56,57]. The primary benefit of such an approach is the fact that it offers a notably long duration of action and could thus be administered in 6-month periods, effectively solving the adherence problem, one of the main impediments in the clinical application of lipid-lowering therapy [58]. The aforementioned ORION-1 trial randomized 501 patients who suffer from ASCVD along with elevated LDL-C levels to receive either inclisiran or placebo. It was demonstrated that participants in the inclisiran group, who were given 300 mg doses, had LDL-C lowered by 52.6% when compared to placebo after a follow-up of 6 months [57]. Furthermore, the ORION-10 and ORION-11 studies demonstrated a reduction by 52.3% and 49.9% for participants—individuals with ASCVD and elevated LDL-C despite maximal statin doses—treated with inclisiran compared to placebo after 18 months of therapy [59]. Additional information will be brought with an ongoing V-DIFFERENCE trial that will examine the effects of inclisiran (300 mg) in around 1700 participants with hypercholesterolemia treated with rosuvastatin [60]. The primary outcome of the V-DIFFERENCE trial is the proportion of participants that achieves individual LDL-C targets (<55 mg/dL or <70 mg/dL) after 90 days. In general, the principal shortcomings of using PCSK9-based treatment in real-life clinical practice include high cost as well as subcutaneous administration with associated injection site reactions [61].

### 3.2. Omega-3 Fatty Acids

The well-known tale of the role of omega-3 fatty acids and fish oil in ASCVD prevention is largely based on epidemiological observations made among Greenlandic Inuit people and in which marked triglyceride reduction was observed [62]. Subsequently, many researchers have challenged the role of omega-3s in this setting, yielding diverse results [63]. The mechanisms underlying the obvious improvement in outcomes are not completely clear. The reduction in serum triglycerides is thought to be a consequence of the hepatic synthesis of triglyceride-rich VLDL, and an additional mechanism is the decrease in their absorption via N-acyl taurines which accumulate in bile following omega-3 supplementation [64]. Apart from that, omega-3 fatty acids likely exhibit additional atherosclerotic protection as well. Specifically, omega-3s were shown to stimulate the production of multiple prostaglandins and lipid mediators that lead to the resolution of tissue injury and inflammation as well as to modulate T-cell differentiation [65]. Furthermore, Tousoulis et al. showed that omega-3 fatty acids improve indices of endothelial function and that they exert anti-inflammatory effects in patients with metabolic syndrome [66]. Nevertheless, not all data are consistent in demonstrating the benefit of omega-3s, which is thought to be a consequence of the type as well as the dosage of omega-3 fatty acids used in the studies [67]. The Cardiovascular Events with EPA—Intervention Trial (REDUCE-IT) trials have shown a ~5% absolute reduction (in comparison to placebo) in a composite endpoint which considered CV death and nonfatal ischemic events for groups who are given 2 g twice daily of icosapent ethyl (IPE), a high-purity prescription form of eicosapentaenoic acid (EPA), whereas the Japan EPA Lipid Intervention Study (JELIS) study showed a clear reduction in MACEs when comparing EPA to placebo [68]. Accordingly, a meta-analysis comprised of 127,477 participants, in which the dose of marine omega-3 supplements and the risk of the specific prespecified outcome were accounted for, demonstrated that omega-3 supplementation was associated with a reduction in myocardial infarction, CV death, and total CV disease [69]. On the other hand, a recent meta-analysis that included 77,917 participants in total showed that consuming marine-derived omega-3 fatty acids over a period of 4.4 years did not lead to a significant decrease in myocardial infarctions or strokes [70]. Overall, the discrepant results in omega-3 benefits lead us to the conclusion that further data are needed before recommending omega-3s for ASCVD prevention and treatment, though the safety profile of omega-3 fatty acids allow us to support of their use in the general population despite a lack of clear benefit.

### 3.3. Bempedoic Acid

Bempedoic acid, a long-chain tetramethyl-substituted keto diacid, is a once-a-day peroral hypolipidemic drug of promising potential [71]. The principal mechanism of its action is the inhibition of ATP citrate lyase (ACLY), an enzyme upstream to the target enzyme of statins (3-hydroxy-3-methylglutaryl-coenzyme A reductase). ACLY is the enzyme positioned at the crossroads of the glucose and lipid metabolism, which explains why its inhibition reduces both de novo cholesterol and fatty acid synthesis [72]. Designed as a prodrug which converts in the liver via very long-chain acyl-CoA synthetase 1 to coenzyme A, bempedoic acid is devoid of effect in tissues which lack this enzyme [73]. The most important tissue in this regard is muscle, which lacks activity of this enzyme, thus explaining the lack of statin-associated myopathy despite the fact that the same pathway is targeted [73]. Such a favorable safety profile concerning adverse events associated to the musculoskeletal system when compared to statins was confirmed by Pinkosky et al. [74]. Early studies demonstrated that bempedoic acid leads to reduction in serum LDL-C levels by increasing LDL-C receptor expression, improvement in hepatic lipids metabolism and glycemic control, reduction in body weight, and attenuation of atherosclerosis [75,76]. Interestingly, bempedoic acid was also shown to possess potent systemic anti-inflammatory effects and to downregulate pathways involved in collagen deposition in the liver [77,78,79]. As they trigger the same pathway, it was speculated that the addition of bempedoic acid to patients whose cholesterol pathways were being inhibited by maximal statin doses would limit its effects [80,81]. Accordingly, a pooled analysis of the available phase 3 trials demonstrated that bempedoic acid monotherapy reduces LDL-C by 17.8% and 24.5% in patients being treated with statins and in patients intolerant to statins, respectively. On the other hand, bempedoic acid monotherapy in statin-naïve patients yielded a 26.5% reduction in LDL-C in comparison to placebo, and when added to ezetimibe (which targets a completely different pathway), the reduction was slightly less than 40% when compared to placebo [82]. Accordingly, Jadhav et al. displayed results which suggested that bempedoic acid added on top of statin therapy is equally effective as high-dose statin monotherapy, yet without the muscular side effects related to high statin doses [83]. Nevertheless, more data must be collected concerning the clinical benefits with respect to ASCVD prevention and treatment prior to recommending bempedoic acid, although several major professional societies have already approved it for patients with established ASCVD, as well as for patients with heterozygous familial hypercholesterolemia [84,85]. Four major clinical studies (CLEAR studies) with follow-up from 12–52 weeks have so far demonstrated the efficacy of bempedoic acid in lowering cholesterol in multiple populations [86,87,88,89]. Furthermore, open label extension of the CLEAR Harmony study showed that bempedoic acid (combined with statins in maximally tolerated dosage) was proven to be both safe and efficacious during the 2.5-year follow-up in patients with hypercholesterolemia and ASCVD with or without familial hypercholesterolemia [90]. On the other hand, anti-inflammatory effects of bempedoic acid were shown to be clinically applicable, as pooled analysis of CLEAR studies demonstrated a significant decrement in hsCRP [91]. In light of existing evidence concerning the role of inflammation in atherosclerosis, one should not neglect these findings. Accordingly, a meta-analysis of phase 2 and 3 randomized controlled trials on bempedoic acid demonstrated its favorable effects on lipid profile and hsCRP levels alongside an acceptable safety profile [92]. Finally, the results of the CLEAR Outcomes study, the first study that investigated effect of bempedoic acid on CV outcomes, was recently published [93]. During a median follow-up of 40.6 months, on a sample of 13,970 statin-intolerant patients, bempedoic acid was shown to significantly reduce MACEs in comparison to placebo (HR 0.87, 95% CI 0.79 to 0.96, *p* = 0.004) as well as the incidence of myocardial infarction and coronary revascularization. Among side effects, the trial confirmed findings from previous studies, as bempedoic acid increased the risk of gout and cholelithiasis in comparison to placebo (3.1% vs. 2.1% and 2.2% vs. 1.2%, respectively). The promising safety and efficacy results of the CLEAR Outcomes trial will probably position bempedoic acid side to side with statins and other lipid-lowering drugs used in ASCVD management.

### 3.4. Pemafibrate

Pemafibrate, a selective PPARα modulator, was suggested to decrease triglyceride levels and raise HDL-C levels [94]. Unlike the US and Europe, in which pemafibrate potential is still being studied in phase 3 RCTs, Japan has approved its use in the treatment dyslipidemia. Available findings imply that pemafibrate leads to a 30% reduction in triglyceride serum levels when used as monotherapy and a 10–15% mean increase in HDL-C after 3 months of treatment. On the other hand, as an add-on therapy for statins, the reduction increased to 50% for triglyceride with a proportional increase in HDL-C. Furthermore, pemafibrate leads to increased hepatic uptake of glucose and improves insulin sensitivity while having fewer side effects than fibrates, a statement which was supported with clinical evidence [95]. Of important note, a quantitative increase in the levels of LDL-C was noticed; however, this was only for medium- and large-sized LDL-C fraction, which may not have the same effect in regard to increasing the risk of ASCVD [96]. In addition, slightly increased homocysteine levels were observed following its use, but since this was noticed in only one study, it is yet to be determined whether such an increase could herald negative clinical consequences through longer periods of time [97]. The PROMINENT trial was designed in order to evaluate cardiovascular outcomes in over 10,000 patients with increased TGs and reduced HDL-C [98]. Participants were randomized into a pemafibrate group and a placebo group in addition to the optimized statin therapy and were followed for 4 years. Even though pemafibrate lowered triglyceride, VLDL cholesterol, remnant cholesterol, and apolipoprotein C-III levels, its use did not lead to a decrease in adverse cardiovascular events. However, despite similar incidence in serious adverse events in comparison to placebo, pemafibrate was linked to an increased occurrence of adverse renal events and venous thromboembolism as well as to reduced incidence of nonalcoholic fatty liver disease. Finally, it is worth mentioning that meta-analysis showed an improved lipid profile in patients taking pemafibrate, which was comparable to that of fenofibrate, but with a lower burden of adverse events [99].

### 3.5. RNA-Based Therapies

The pharmacological targeting of messenger RNA represents an innovative approach to treat ASCVD. ISIS-APO(a)_Rx_ is a new antisense oligonucleotide intended to bind to apo(a) mRNA with triantennary N-acetyl galactosamine in order to preclude translation, which consequently stops the production of apo(a) and the decreasing of Lp(a) levels [100,101]. Following promising results in animal studies, a phase 1 clinical study confirmed a decrease of Lp(a) by up to 92% (mean 78%) after 36 days, and the only observed side effects were related to the injection site [102,103]. It is also worth noting that the formulation was equally effective across all isoforms and was not dependent on the baseline Lp(a) levels. Furthermore, a recent phase 2 trial with olezarsen, a conjugated antisense oligonucleotide targeted to hepatic APOC3 mRNA in order to inhibit apoC-III production, demonstrated that olezarsen significantly reduced apoC-III, triglycerides, as well as atherogenic lipoproteins in a population with moderate hypertriglyceridemia who are either at high risk of or have established ASCVD [104]. Finally, several phase 3 trials are currently exploring the effects of antisense oligonucleotides on the treatment of dyslipidemia. Specifically, a phase 3 trial is exploring the effects of olezarsen in patients with familial chylomicronemia syndrome [105]. A separate and phase 2/3 trial is exploring the effects of volanesorsen in patients with hypertriglyceridemia and familial partial lipodystrophy, whereas a phase 3 trial aims to determine the effects pelacarsen in patients with established ASCVD and Lp(a) levels of >70 mg/dL [106,107].

### 3.6. Therapies Based on Apolipoprotein A-I

Apolipoprotein A-I is considered to be the main component of HDL particles and is accountable for the cholesterol efflux capacity [108]. After an acute myocardial infarction, there is a high likelihood of recurrent MACE (around 12% within one year of the index event) [109]. It is believed that reverse cholesterol transport (RCT), which is commonly impaired in the setting of myocardial infarction, might explain recurrent cardiovascular events in these patients, as impaired RCT may lead to further cholesterol accumulation in the arterial wall [110]. In consideration of the role of HDL in this process, human plasma-derived apolipoprotein A-I (CSL112) was assessed in the ApoA-I Event Reducing in Ischemic Syndromes (AEGIS) I trial [108]. The results of the AEGIS I trial demonstrated the feasibility and the renal and hepatic safety of CSL112. On the other hand, AEGIS II is an ongoing trial which aims to demonstrate whether CSL112 will improve outcomes in high-risk patients with acute myocardial infarction [111]. Other therapies based on apoA-I are also available, and these are currently being explored in phase 2 trials [112,113]. Apart from apoA-I infusion therapies, cholesteryl ester transfer protein, an important enzyme in HDL metabolism, also emerged as a viable option for ASCVD management. So far, the only cholesteryl ester transfer protein inhibitor which has demonstrated benefit as an add-on therapy to statins is anacetrapib [114].

### 3.7. Immunomodulatory Therapies

Anti-inflammatory drugs such as canakinumab, colchicine, and methotrexate have been extensively studied in many clinical trials, yet none of these trials have focused on potential CV benefits [115]. Nevertheless, in recent years, a substantial amount of evidence has implicated inflammation in the pathogenesis and progression of atherosclerosis, and this has inspired researchers to explore many inflammation-associated targets. The principal study in this regard is the Canakinumab Anti-inflammatory Thrombosis Outcome Study (CANTOS), in which the CV effects of monoclonal antibodies towards IL-1β were explored in a population of patients with previous myocardial infarction and hsCRP ≥ 2 mg/L [116]. Canakinumab was shown to reduce the incidence recurrent cardiovascular events more than placebo, independent of lipid-level lowering, but at the price of a higher incidence of infections and fatal infections. Unsurprisingly, a secondary analysis of the CANTOS trial demonstrated that the extent of CV benefit was heralded by a reduction in hsCRP following treatment with canakinumab [117]. On the other hand, colchicine, a well-established medication in terms of treating gout and pericarditis, was explored in the Colchicine Cardiovascular Outcomes Trial (COLCOT) [118]. Low-dose colchicine (0.5 mg/day) in patients with recent myocardial infarction led to a significantly lower risk of ischemic cardiovascular events when compared to placebo, yet this was accompanied by an observed increase in the occurrence of pneumonia. The Low-Dose Colchicine 2 (LoDoCo2) trial yielded consistent results in patients with chronic coronary disease [119]. Furthermore, a recent study based on CT-coronary angiography demonstrated a decrease in low-attenuation plaque volume and hsCRP serum levels after 1 year of follow-up [120]. However, the biggest impediment to the successful implementation of colchicine in ASCVD management algorithms is colchicine-associated gastrointestinal distress, which causes drug discontinuation in as much as 10% of patients [121]. Although early reports suggest that colchicine inhibits the microtubular function of leukocytes, it is worth noting that we are still not completely sure in which ways colchicine exerts its anti-inflammatory effects [122,123,124]. The potential of methotrexate (target dose of 15 to 20 mg weekly) in ASCVD was evaluated in the Cardiovascular Inflammation Reduction Trial (CIRT) [125]. The CIRT Trial yielded disappointing results and was in fact stopped by the Data and Safety Monitoring Board for futility after a median of 2.3 years. When compared to placebo, low-dose methotrexate did not lower IL-1β, IL-6, or hsCRP, yet it led to a reduction in hematocrit and WBC count as well as an increase in liver enzymes and incidence in of non-basal-cell skin cancers.

On the other hand, the recently recognized role of adaptive immunity in pathophysiology of atherosclerosis has opened a new panel of possible therapies. Preclinical studies indicate that IgM antibodies that recognize epitopes associated with oxLDL or pathogens can limit the progression of atherosclerosis [126]. Based on these findings, the use of immunogenic epitopes of apolipoprotein B has received attention from several groups, although, considering the large discrepancy between humans and rodents in terms of immune system, it is worth noting that many obstacles have to be overcome in order to implement these therapies [127,128,129]. On the other hand, a phase 2/3 trial is currently examining the safety of low-dose IL-2 in patients with acute coronary syndromes [130].

## 4. Future Perspectives and Conclusions

LDL-C being the “usual suspect” of atherosclerosis formation and progression, stringent lipid-lowering therapy is perhaps the key to preventing unfavorable outcomes arising as a consequence of atherosclerosis. Statins and adaptations of healthy lifestyle have so far served the purpose in this regard, but targeting other pathways that promote atherosclerosis through dyslipidemia could offer healthcare providers significantly more tools to improve patient outcomes. Finding new targets is particularly warranted, as LDL-C target goals in ASCVD prevention have progressively become lower over the decades with the ever-increasing prevalence of obesity, diabetes, and metabolic syndrome worldwide [131]. Patient groups that will presumably benefit the most from such alternative or supplemental therapies are those who are unable to take statins owing to significant side effects or who still have inappropriately high LDL-C despite a maximal tolerated dose of statins. Among the novel arsenal of drugs in ASCVD management, PCSK9-based drugs (PCSK9 inhibitors and inclisiran) and bempedoic acid seem to be the most promising. Aside from an extremely favorable safety profile, the biggest advantages of PCSK9-based drugs are their massive lipid-lowering potential and a pharmacokinetic profile that enables periodic dosing. On the other hand, the primary incremental benefit of bempedoic acid is the fact that a combination of ezetimibe and bempedoic acid represents a relatively cheap but equally effective alternative for statin-intolerant patients. In fact, a recent analysis suggested that the addition of bempedoic acid would reduce the projected need for PCSK9 inhibitors and the treatment cost of lipid-lowering therapy, with particular benefit for statin-intolerant patients [132]. The emerging field of targeting inflammation (even with “older” drugs such as colchicine) might also prove to be a cost-effective way of managing ASCVD, especially if safety issues are properly dealt with. In addition, the recently recognized role of hematopoiesis in atherosclerosis will enable us to reveal previously undisclosed pathways that promote the process of atherogenesis, which will hopefully provide us with a completely new panel of therapeutic targets.

To conclude, the critical objective in future ASCVD management is to embrace emerging evidence in the field of atherosclerosis, because clinicians are often burden by common practice and personal experience, which have so far been shown to be futile in the setting of atherosclerosis. In this regard, perhaps the most important change in perspective to be noted is the fact that this is not a degenerative continuous process that comes with aging, but is rather an episodical process which can stay dormant for many years and then rapidly progress, but which can also regress, especially upon adequate implementation of lifestyle/therapeutic interventions, especially lipid-lowering strategies.

## Data Availability

Not applicable.
